# A Pathophysiological Model of Parkinson’s Disease Based on Microvascular Flow Disturbance and Leukocyte-Mediated Oxidative Injury in Critical Pigmented Neuronal Niches

**DOI:** 10.3390/antiox15020201

**Published:** 2026-02-03

**Authors:** Emilio Fernández-Espejo, Fernando Rodríguez de Fonseca

**Affiliations:** 1 Royal College of Physicians of Seville, 41013 Seville, Spain; 2 Unidad Clínica de Neurología, Instituto de Investigación Biomédica de Málaga & Plataforma en Nanomedicina-IBIMA Plataforma BIONAND, Hospital Regional Universitario, 29590 Málaga, Spain

**Keywords:** Parkinson’s disease, model, blood flow disturbance, neuromelanin, lipofuscin, leukocyte, myeloperoxidase, dopamine oxidation, alpha-synuclein

## Abstract

The authors hypothesize that idiopathic Parkinson’s disease may result from an alteration in microvascular flow at a “critical point” in the nervous system that is characterized by pigmented cells that express neuromelanin and/or lipofuscin. “Critical points” include the olfactory epithelium/bulb, the autonomic nervous system, the enteric nervous system, the prefrontal–cortico-pontine network, and the amygdala. Hypoxia–ischemia following blood flow disturbance would recruit and activate leukocytes and induce the infiltration of peripheral immune cells into neural tissue. The excess of toxic factors produced by hyperactive immune cells, such as myeloperoxidase and its derivatives, would cause the oxidation of lipids, proteins, and biogenic monoamines such as dopamine, which in turn would facilitate the accumulation and precipitation of neuromelanin, lipofuscin, and alpha-synuclein. In addition, neuromelanin and lipofuscin precipitates may accentuate the misfolding and aggregation of alpha-synuclein. This “amplification” mechanism could help explain the crucial role of pigmented neurons in the onset of Parkinson’s disease pathology, triggering abnormal neurotoxic alpha-synuclein spread throughout the nervous system from the “critical point” of origin, and enabling a self-perpetuating degenerative process. The proposed hypothesis may have implications for the identification of new therapeutic targets, early prevention strategies, and the development of vascular and/or immune biomarkers.

## 1. Introduction

Parkinson’s disease (PD) is characterized by the heterogeneity of symptoms and the involvement of different neuronal systems during the early phases of the disease. Borghammer et al. proposed that the origin of PD pathology could be explained by two models: the peripheral nervous system first (PNS-first model) and the central nervous system first (CNS-first model) [1,2,3]. Other authors propose a multifocal model [4,5,6]. The PNS-first model, also known as body-first, is characterized by marked damage to the PNS, including the autonomic nervous system (ANS) and/or the enteric nervous system (ENS), prior to nigrostriatal damage. The PNS-first model is based on the studies of Braak et al. who analyzed, in PD patients, the distribution of inclusions of alpha-synuclein (αSyn), in the form of Lewy bodies (LBs) and Lewy neurites (LNs), within the nervous system [7,8]. They describe that the initial phase of the disease (stage 1) is characterized by the identification of αSyn aggregates in the dorsal motor nuclear complex of the glossopharyngeal and vagus nerves (DMC) in the brainstem, as well as in the olfactory bulb and anterior olfactory nucleus [7,8]. Since visceromotor projections connecting the vagus nerve and DMC are also affected at this stage, enteric nerve plexuses often show αSyn pathology [9,10,11,12,13,14,15,16,17,18,19,20,21,22]. Furthermore, the ANS, including cardiac sympathetic nerves and autonomic ganglia, is frequently affected with αSyn pathology [9,10,11,12,13,14,15,16,17,18,19,20,21,22]. In fact, it is proposed that the disease may start in the periphery of the nervous system before stage I. The pontine reticular nucleus and the locus coeruleus are frequently damaged, the reason why the PNS model is much associated with the presence of isolated REM sleep behavior disorder (iRBD) during the early phases of the disease [1], as well as early symptoms of depression and anxiety preceeding the motor disturbances [23]. IRBD is considered as a prodromal stage of PD and other α-synucleinopathies such as dementia with Lewy bodies and multiple system atrophy. The spread of αSyn to other regions of the nervous system would occur over time due to neuron-to-neuron transmission [24], following Braak’s staging model [7,8].

On the other hand, some patients (7–43% according to the author) do not fit Braak’s sequence, indicating the existence of a different pathogenic model. This model is called the “CNS-first” or “brain-first” model because it is characterized by pronounced damage to CNS areas such as the nigrostriatal circuit or the amygdala before involvement of the PNS. For example, Kalaitzakis et al. [25] studied 71 cases of PD, and although the substantia nigra was affected in 100% of cases, the DMC was not affected in 7% of cases. Parkkinen et al. [26] studied 226 αSyn-positive subjects, and some subjects (17%) deviated from the proposed caudo-rostral spread according to Braak’s staging model. Zaccai et al. [27] examined 66 brains with Lewy bodies, and half (51%) conformed to Braak’s hypothesis, while 17% lacked pathology in a lower brainstem region. This group of patients develops motor and cognitive impairments without premotor autonomic/enteric dysfunction, and DMC is not damaged early [6,25,26,27,28,29,30,31].

Finally, a possibility is that both the peripheral and central neuronal systems are damaged from the onset of the disease [6]. This so-called “blood-borne mechanism hypothesis” is based on the existence of a selective cellular vulnerability to a blood-borne neurotoxin and subsequent immunological reactions leading to mitochondrial dysfunction and multicentric damage of catecholaminergic neurons [4,5,6,32]. A lower functional threshold for non-dopaminergic neurons would help explain why non-motor symptoms, including sleep disturbances, depression and anxiety, emerge earlier than motor symptoms (that are associated with death of nigrostriatal dopaminergic neurons).

## 2. Site of Origin of Parkinson’s Disease: Pigmented Cells May Play a Crucial Role

### 2.1. Critical Points

Although some authors indicate that LBs/LNs have so far not been described “only in the gut”, “only in the heart” or “only in the olfactory bulb” before the onset of clinical PD [11,12,13], others have reported the presence of αSyn inclusions in incidental Lewy body disease (ILBD). This disease is considered as a prodromal stage of PD and is characterized by the post-mortem finding of LBs in the brains of people without clear symptoms of PD or Dementia with Lewy Bodies during life. Thus, there are reports of LBs/LNs in ILBD confined to the anterior olfactory nucleus, olfactory bulb, vagus nerves, myenteric plexus of the esophagus and intestine, cardiac sympathetic nerves, peripheral autonomic ganglia, submandibular gland, adrenal medulla, DMC, pontine nuclei, amygdala, sacral and thoracic medulla, and autonomic nerves of numerous organs such as skin, retina, kidney, etc. [9,13,14,15,16,17,18,19,20,21,22,33].

**Figure 1:**
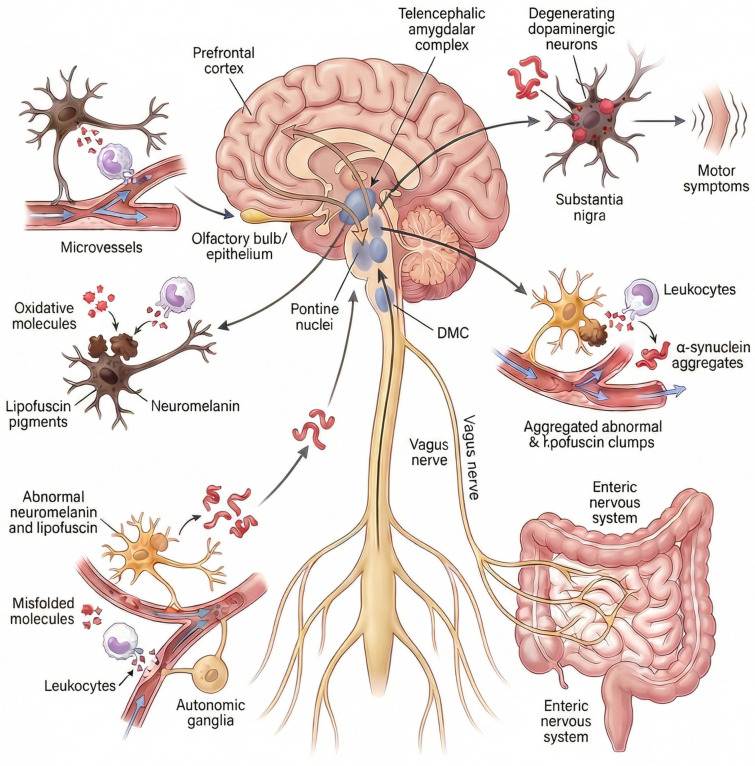
Hypothesis on the site of origin of idiopathic Parkinson’s disease. Idiopathic Parkinson’s disease is caused by microvascular flow disturbance in a “critical point” of the nervous system. The “critical points” are specific pigmented neuronal niches located in the olfactory epithelium/bulb, the peripheral nervous system/brain axis, the prefrontal–cortico-pontine network, and the amygdala [9,12,14,15,16,17,18,19,20,21,22]. The peripheral nervous system comprises the autonomic nervous system (autonomic ganglia, cardiac sympathetic nerves, autonomic nerves of numerous organs, such as skin, retina, adrenal glands, kidneys, etc.) and the enteric nervous system or ENS (the Auerbach/Meissner plexuses of the esophagus and intestine). The ENS connects with the DMC via the vagus nerves. The prefrontal-cortico-pontine network comprises the prefrontal cortex in connection with pontine nuclei such as the locus coeruleus and the pontine tegmental reticular nucleus. Pigmented neurons express the pìgments neuromelanin or lipofuscin. Microvascular flow alteration and leukocyte-mediated oxidation induce the accumulation and precipitation of abnormal neuromelanin, lipofuscin and alpha-synuclein. Neuromelanin is expressed in the olfactory epithelium/bulb, the pontine nuclei, the DMC, the sympathetic ganglia, and the amygdala. Lipofuscin is progressively expressed in neuronal cells in association with aging, mainly by neurons in the Auerbach/Meissner plexuses of the gut, isocortex, basal nuclei of the brain, ganglion cells, and nerves of various organs, such as the heart, retina, adrenal glands, and kidneys. Pathological, neurotoxic alpha-synuclein species propagate throughout the nervous system from these critical points Via a self-perpetuating, prion-like mechanism, progressively accumulating within vulnerable neuronal populations. The convergence of this pathology in dopaminergic neurons of the substantia nigra pars compacta ultimately leads to their degeneration and the emergence of cardinal motor symptoms. Abbrev.: DMC, dorsal motor nuclear complex of the glossopharyngeal and vagus nerves.

It is important to note that Beach et al. [33] analyzed cases of ILBD in which a single area contained LBs/LNs to establish the main region initially affected by PD. The olfactory bulb was the most frequently affected brain region in isolation (approximately 15% of cases), followed by the medulla oblongata (that includes the DMC) and the pons (~12% of cases each). In addition, less than 4% of cases showed pathology in the amygdala. Interestingly, in this study, there were no cases of clinical PD affecting only a single brain region. Therefore, available postmortem evidence supports that the remaining brain regions involved in PD are affected after specific “critical points”: the olfactory bulb, the DMC (connected to the ENS), the pontine nuclei, or the amygdala (Figure 1).

The spread of misfolded αSyn to other regions of the nervous system would occur over time following a mechanism similar to that of prions, by which pathological conformers of αSyn act as self-propagating templates that induce misfolding, aggregation, and fibrillation of native, soluble αSyn in recipient neurons, leading to progressive amplification of toxic species and anatomically ordered propagation along vulnerable neuronal networks. The convergence of this pathology in dopaminergic neurons of the substantia nigra pars compacta ultimately leads to their degeneration and the emergence of cardinal motor symptoms, thereby explaining why diverse non-motor clinical manifestations may precede the diagnosis of PD.

### 2.2. Neuromelanin and Lipofuscin

It should be noted that there is a common factor among all “critical points”: they are anatomical niches containing pigmented cells that express neuromelanin (NM) and/or lipofuscin (LF). These pigments are related to cellular vulnerability in PD [34,35,36]. Both pigments originate from incompletely degraded proteins and/or lipids derived from products of oxidized catecholamines or the breakdown of lysosomes and mitochondria, processes that are involved in the pathology of PD [35,36,37]. Furthermore, aging is a risk factor for PD, and NM and LF are considered age-related pigments because, with aging, there is accumulation of vacuoles filled with NM and LF in cells [36,38]. These pigments are central to the lysosomal-mitochondrial axis theory of aging and neurodegenerative diseases [36,37].

Regarding the presence of NM at “critical points”, it is normally expressed by neurons in the locus coeruleus, the substantia nigra, the DMC, other pontine nuclei, and the spinal and sympathetic ganglia [39,40,41,42,43]. Pigmented cells expressing NM have been found in the olfactory epithelium and olfactory bulb [44]. On the other hand, the amygdala is frequently affected (along with the pontine nuclei) by melanin-related pathologies, such as neurocutaneous melanosis [45,46]. NM is also present in glial cells in PD, but this pigment appears to be phagocytosed by glial cells after its release from degenerated NM-containing neurons [47].

As for LF and “critical points,” LF is progressively expressed in neuronal cells in association with aging, mainly by neurons in the Auerbach/Meissner plexuses of the gut, isocortex, basal nuclei of the brain, ganglion cells, and nerves of various organs, such as the heart, retina, adrenal glands, and kidneys [35,36,48,49]. LF expression in the brain increases significantly in PD and is considered a determining factor in this neurodegenerative disorder [35,50]. Interestingly, it has been proposed that the accumulation of LF in neurons of the isocortex, including the prefrontal cortex, marks senescence in humans [51].

## 3. Hypothesis on PD Onset Based on Microvascular Flow Disturbance

It can be hypothesized that idiopathic PD may be related to the occurrence of pathological events at “critical points” of the nervous system. In this regard, several studies with iRBD patients are suggestive of the occurrence of microvascular flow disturbances and hypoperfusion in the brain before the onset of PD. As previously explained, iRBD is a prodromal stage of Parkinsonism, and the estimated risk of iRBD progressing to α-synucleinopathy is 90.9% after 14 years [52,53,54,55,56]. Alteration in microvascular blood flow could represent the decisive event associated with the onset of PD.

Cortical hypoperfusion and microvascular flow disturbances are observed in patients with iRBD throughout the cortex [57], and the frontal cortex is always reported as affected area [57,58]. Alterations in microvascular flow are detectable before any cerebral atrophy and are thought to be caused by loss of neurogenic control of blood flow due to previous degeneration of monoaminergic (noradrenergic, dopaminergic, and serotonergic) innervation [57]. Thus, noradrenergic fibers from the locus coeruleus innervate parenchymal microvessels throughout the brain [58,59,60]. Dopaminergic fibers originating in the substantia nigra and ventral tegmental area project to microvessels within the prefrontal, cingulate and entorrhinal cortices [61,62,63,64]. Serotonergic fibers from the raphe nucleus innervate parenchymal capillaries throughout the cortex and the hippocampus [65]. The cholinergic innervation from the nucleus basalis of Meynert may be involved as well [61]. To sum up, degeneration of pontine nuclei, the substantia nigra, and the nucleus basalis of Meynert would impair flow regulation in the cerebral cortex in patients with iRBD.

Although microvascular hypoperfusion in iRBD throughout the cortex appears to be a secondary phenomenon, i.e., a response to the neurodegenerative protein pathology present in neurons, the authors speculate on the possibility that a critical aspect of the pathological process manifests itself in reverse. Therefore, microvascular flow disturbance, whatever the mechanism, in a critical cortical area may appear first. One critical region may be the prefrontal cortex, which is a common locus showing hypoperfusion in patients with iRBD and clinical PD [56,57,66,67,68,69]. Microvascular flow alterations and hypoperfusion within the prefrontal cortex, a region where LF accumulates with age [51], would induce retrograde damage to pigmented neurons in the pons. This damage may involve axonal degeneration along with metabolic, inflammatory, and redox dysfunctions. The prefrontal cortex is connected to the pontine nuclei, primarily the basilar pontine gray matter, the pontine tegmental reticular nucleus, caudal and oral pontine reticular nuclei, and the locus coeruleus [70,71], and most of them are rich in catecholaminergic and NM-containing neurons.

**Figure 2:**
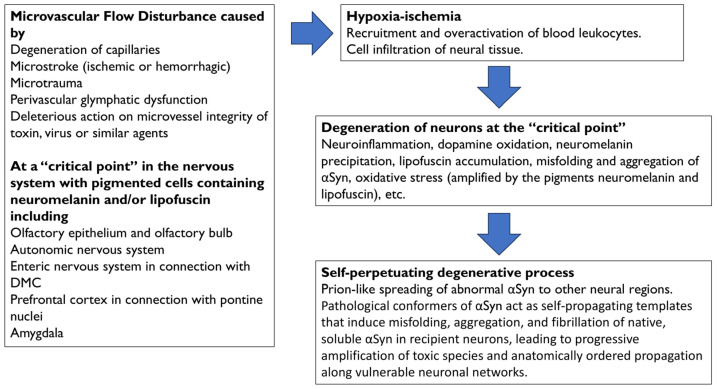
Physiopathology events that are associated with the onset and progression of Parkinson’s disease. Microvascular flow disturbances at “critical points” of the nervous system are caused by degeneration of capillaries, microstroke (ischemic or hemorrhagic), microtrauma, perivascular glymphatic dysfunction, or the deleterious action on microvessel integrity of toxins, viruses, or similar agents. Specific parkinsonian vulnerability of “critical points” may be associated with the presence of neuromelanin and/or lipofuscin. Hypoxia–ischemia following hypoperfusion would recruit leukocytes and other immune cells, which would infiltrate the neural tissue. Toxic factors derived from hyperactive leukocytes, such as myeloperoxidase and derivatives, would cause neuroinflammation, oxidative stress, dopamine oxidation, oxidation and precipitation of neuromelanin, lipofuscin formation and accumulation, and misfolding and aggregation of αSyn. Oxidative stress is amplified by the accumulation of the pigments neuromelanin and lipofuscin. The degeneration of brain nuclei would be followed by the spreading of αSyn to neighboring regions, and a self-perpetuating degenerative process would be triggered. Abbrev.: αSyn, alpha-synuclein; DMC, dorsal motor nuclear complex of the glossopharyngeal and vagus nerves.

A summary of the degenerative events following microvascular flow alteration in the prefrontal cortex, as shown in Figure 2 and further explained in the next section, is as follows: (a) Hypoxia–ischemia following hypoperfusion would recruit leukocytes and other immune cells, which would become hyperactive and infiltrate neural tissue; (b) the excess of toxic factors released by infiltrated cells, such as myeloperoxidase and its derivatives, would cause neuroinflammation, oxidative stress (amplified by LF accumulation), cell death with leakage, oxidation, and precipitation of NM, as well as the misfolding and aggregation of αSyn, within cortical terminals and neurons of the pontine nuclei; (c) the degeneration of neurons within the pontine nuclei would be followed by spread of abnormal αSyn to neighboring regions, and (d) a self-propelling degenerative process would be triggered.

A similar sequence of events may occur at each “critical point,” and the authors propose that leukocyte infiltration may be largely localized to a single “critical point” in each case of PD. Leukocyte-mediated oxidative stress in pigmented cells would trigger localized neuronal degeneration at the “critical point,” followed by the propagation of the anomalous neurotoxic αSyn from the point of origin.

It is true that there is no evidence to support the idea that PD patients may experience decreased perfusion due to flow disturbances, microinfarcts, or similar conditions decades before PD diagnosis, apart from the mentioned changes in the prefrontal cortex in patients with iRBD. A study needs to be conducted in newly diagnosed PD patients who have undergone brain magnetic resonance imaging (MRI) or single photon emission computed tomography (SPECT) between 5 and 20 years prior to diagnosis for other reasons. These imaging studies could be quantified to detect the presence of microvascular lesions in the prefrontal cortex or amygdala and compared with a matched control group. Such a study could support or refute the current hypothesis. A revision of published studies in presymptomatic patients using SPECT could provide interesting data as well. On the other hand, microvascular flow alterations and epistaxis (frequently caused by environmental toxins) are common in the olfactory epithelium [72], which is closely connected to the olfactory bulb. It is clearly more difficult to demonstrate microvascular flow alterations in other critical areas, such as the enteric nervous system, years before the diagnosis of PD.

## 4. Data Supporting the Involvement of Microvascular Flow Disturbance, Leukocyte Trafficking, and Leukocyte-Mediated Oxidation in PD Pathology

### 4.1. Microvascular Flow Disturbance and Vulnerability of Pigmented Neurons to Perfusion Deficit

Alteration of microvascular flow may be associated with vascular pathologies, such as degeneration of capillaries, microstroke (ischemic or hemorrhagic), microtrauma, perivascular glymphatic dysfunction, or deleterious action on microvessel integrity of toxins, viruses, or similar (Figure 2). It is worth clarifying how the microvascular flow disturbance proposed as the cause of PD pathology differs from other vascular neurological pathologies, such as vascular dementia or vascular parkinsonism. An important basis for the proposed hypothesis is that microvascular flow disturbance occurs at a single “critical point,” unlike what occurs in multi-infarct vascular dementia. In the proposed scenario, it is possible that only a few microvessels are affected and that these are responsible for cell damage. In this context, it is known that even a single microstroke can cause cell death in the ischemic region [73,74]. The proposed flow disturbance leads to Lewy body pathology due to leukocyte-mediated oxidation, but since the affected area is small, cavitations are not observed. There may be small lesions in the white matter, and only MRI studies could confirm this. On the other hand, vascular parkinsonism has different characteristics from the proposed model. This type of parkinsonism is frequently associated with multiple strokes or microangiopathies affecting the basal ganglia, identified by brain scans showing changes in the white matter or infarcts. Finally, vascular dementia and vascular parkinsonism may be caused by cerebral small vessel disease, a vascular disease characterized by multiple lacunar infarcts and microbleeds that cause damage to the Blood–Brain Barrier (BBB) [75,76,77,78].

Alteration of microvascular blood flow is frequently found in PD apart from the aforementioned disturbance in cortical blood flow. Thus, microvascular abnormalities and decreased perfusion in the retina of patients with PD are detected in the early stages of the disease [79,80]. Furthermore, although these alterations may be due to advanced disease, altered morphology and degeneration of capillaries and cortical microinfarcts are common findings in brain autopsies of individuals with age-related degenerative diseases [81,82,83]. Perivascular alterations are associated with dysfunction of the glymphatic system and the accumulation of αSyn aggregates in the dilated perivascular space in patients with PD [84]. Furthermore, neurovascular alterations can cause aberrant angiogenesis [75,85] and BBB damage [85], phenomena frequently observed in mesencephalic regions of PD patients [86]. Finally, microvascular flow alterations are common in the olfactory epithelium, and external factors such as toxins or viruses may induce microvascular damage followed by Lewy pathology in the olfactory bulb or enteric nervous system.

In addition, neurons implicated early in PD, including prefrontal cortex and amygdala neurons, dopaminergic neurons of the substantia nigra pars compacta, and noradrenergic neurons of the locus coeruleus, are uniquely vulnerable to such subthreshold perfusion deficits. These neurons exhibit exceptionally high metabolic demand, extensive axonal arborization, reliance on autonomous pacemaking, and elevated mitochondrial oxidative stress. The accumulation of neuromelanin and redox-active catecholamines further amplifies susceptibility to metabolic stress without immediate cell lysis. In the case of LF accumulation, this pigment contributes to chronic oxidative stress and impaired cellular clearance. Formed through iron-catalyzed lipid and protein oxidation, LF is closely linked to mitochondrial reactive oxygen species (ROS) production [35,36,37]. Once accumulated, it resists lysosomal degradation, occupies lysosomal volume, and reduces autophagic and proteolytic efficiency. This lysosomal dysfunction impairs mitochondrial quality control, allowing damaged mitochondria to persist and further amplify oxidative stress. Experimental evidence shows that LF can directly induce mitochondrial generation of reactive oxygen species and lysosomal instability, establishing a feed-forward cycle of oxidative injury. In neurons, which rely heavily on proteostasis and mitochondrial function, this process imposes a sustained energetic and proteostatic burden rather than causing acute cell death, making LF-loaded neurons vulnerable to subthreshold hypoperfusion.

### 4.2. Role of Leukocytes

Following microvascular damage, subsequent hypoxia–ischemia would induce the activation and mobilization of blood leukocytes [87,88,89,90], that accumulate near the damaged tissue, enter the neural tissue, and release myeloperoxidase (MPO) and other oxidative and proinflammatory factors [89,90,91,92]. As a result, glial cells are activated in the brain, microglia in the olfactory bulb, or enteric glial cells in the gut, as appropriate, which in turn would release more MPO and cytokines causing further leukocyte trafficking and transmigration [75,93]. BBB injury may also facilitate leukocytes to transmigrate to the brain. Redox imbalance would play a central role as the unifying driver of these processes.

The presence of peripheral immune cells in the brain is a key factor in PD and other neurodegenerative diseases [94]. It is known that when the BBB is disrupted by cerebral ischemia, peripheral immune cells, including lymphocytes, monocytes, and neutrophils, can enter the CNS, where they mediate proinflammatory and oxidative effects [95]. Regarding the type of leukocyte involved in PD pathology, transcriptomic and genetic studies point to monocytes as the critical cell, and higher count of monocytes is detected in the cerebrospinal fluid (CSF) of PD patients [95]. However, it remains a difficult question to study whether or not these cells invade the brain parenchyma at any point in PD [96]. Since infiltrated monocytes differentiate into tissue-specific macrophages (which also contain MPO), the homeostatic genetic signatures that could distinguish monocytes from other myeloid cells are rapidly lost after leaving the circulation [97]. However, several studies in patients with PD or in postmortem tissue indicate that there is a peripheral innate immune response that depends mainly on the activation and trafficking of blood monocytes. For example, the monocyte population in PD is abnormal, hyperinflamed, infiltrates the brain, and contributes to disease progression [91,92,95,98,99]. Gene expression studies show that the prefrontal cortex (one of the critical points proposed in this article) in PD patients has a higher number of monocytes compared to healthy controls, with no differences in other immune subtypes [99]. Finally, Gellhaar and colleagues report the presence of MPO-positive blood-derived cells (probably macrophages) in the vascular wall or neighboring parenchyma of the nigrostriatal regions of postmortem tissue from PD patients [100].

### 4.3. Role of Myeloperoxidase-Derived Products, NM, LF, and Catecholamines

Leukocyte-MPO activity and concentration in serum are increased in PD [101,102], and hyperactive leukocytes release excess hypochlorite, reactive oxygen species, reactive nitrogen species, and other chlorinated compounds, which are potent proinflammatory and oxidative agents that damage dopaminergic neurons [88,103,104,105,106,107,108,109]. Hypochlorite and reactive species induce oxidative modifications of lipids, biogenic amines such as dopamine, and proteins, leading to misfolding and aggregation of proteins such as αSyn, hallmark of PD [106,109,110,111,112,113]. Hypochlorite reacts with dopamine at physiological concentrations to form neuromelanin deposits [109], and NM precipitation would exacerbate αSyn aggregation [114,115,116]. It should be noted that a key factor in the pathology of PD is that both oxidized αSyn and NM precipitate in the form of toxic aggregates [114,116,117]. In addition, hypochlorite and reactive oxidative species can cause degradation of lysosomes and mitochondria, oxidation of catecholamines, and activation of microglia, all of which are processes closely related to the formation of NM and LF deposits [35,36,50,111,112,118,119,120]. The accumulation of LF may also exacerbate the misfolding and precipitation of αSyn [116]. Thus, in animal models of PD, the more LF deposits there are, the greater the accumulation of αSyn in limbic and nigral cortical neurons [121].

According to the proposed hypothesis, in which niches of pigmented neurons play a crucial role in the pathology of PD, it appears that NM and LF critically enhance the oligomerization and aggregation of αSyn and, therefore, could facilitate the formation of LBs/LNs. Furthermore, the death of pigmented neurons in PD causes the leakage of oxidized MN into the extracellular space, which may activate microglia, which in turn may induce LF formation and aggregation and further dopaminergic neurodegeneration [114,119,122]. The selective vulnerability of catecholaminergic neurons in PD could also be explained, apart from vulnerability to hypoperfusion, by the fact that oxidized catecholamines are a major source of NM and LF deposits. The harmful mechanism accentuated by cellular pigments can be “triggering.” In other words, the pigment-based mechanism would be similar to “lighting a fuse and the resulting explosion,” with the explosion referring to the accumulation and spread of αSyn.

In summary, peripheral or central pigmented cells containing NM and/or LF at “critical points” in the nervous system would be exposed to the harmful effects of MPO-derived hypochlorite, reactive oxygen species, and other neurotoxic molecules. The combined action of microvascular flow disturbance, leukocyte-mediated oxidation, glial cell activation, redox imbalance, and neurotoxic factors, together with the selective reactivity of hypochlorite and reactive species with NM, LF, αSyn, and catecholamines such as dopamine, would be behind the pathology of Lewy bodies.

### 4.4. Genetic Data

Genetic studies support the involvement of vascular dysfunction and leukocyte-based innate immune response in PD [123,124]. Of the nearly 100 genes identified by genome-wide association studies (GWAS), approximately 10% of the variants are related to monocyte or granulocyte activation/migration/adhesion (*FCGR2A*, *MED12L*, *CLCN3*, *FYN*, *RPS12*, *FAM49B*, *GBF1*, *NOD2*, *FAM171A2*). In addition, *LRRK2* and *PINK1* genes, mutations of which are frequently associated with inherited PD, are related to monocyte activity [124,125,126], phagocytosis [125,126], and vascular cell adhesion [127]. Missense mutations in glucocerebrosidase gene (*GBA1*) are associated with an increased risk of PD, and glucocerebrosidase activity is significantly reduced in monocytes from PD patients [128]. The genes *FYN*, *FAM49B*, and *FAM171A2* are also linked to vascular dysfunction, pointing to a prominent role of these three variants in PD pathogenesis. The proposed vascular-immune-oxidative axis is not intended to exclude other pathogenic pathways, but rather to act as an initiating or amplifying mechanism within a multifactorial disease framework.

## 5. There May Be Four Subtypes of PD

Four subtypes of PD can be defined according to the site of microvascular flow disturbance: olfactory epithelium/bulb PD, PNS/brain PD, prefrontal–cortico-pontine PD, and amygdaline PD (Figure 1, Table 1). It should be noted that these subtypes are intended as a theoretical framework rather than a clinical or diagnostic classification. The subtypes are as follows:

(a) Olfactory epithelium/bulb PD, where the starting point is the microvascular system of the olfactory epithelium that is connected to the olfactory bulb. Both olfactory structures contain pigmented cells. The abnormal αSyn formed in these structures would spread to the anterior olfactory nucleus and secondary olfactory areas before invading the supratentorial and brainstem regions [7,8,10,22,129,130].

(b) PNS/brain PD, where the origin of the disease is the periphery of the nervous system, and the misfolded αSyn would spread throughout the nervous system following Braak’s staging model. The PNS encompasses the ANS, connected with the autonomic areas of the brain, and the ENS, connected with the DMC. The ANS includes the sympathetic ganglia, cardiac sympathetic nerves, submandibular gland, adrenal medulla, and autonomic nerves of numerous organs such as the skin, retina, kidneys, etc. [9,10,11,12,13,14,15,16,17,18,19,20,21,22,130,131,132,133]. The ENS mainly encompasses the Auerbach/Meissner plexuses of the esophagus and intestine. The DMC, adrenal medulla, and sympathetic ganglia are rich in NM pigmented cells. LF is expressed in neurons of the ENS, isocortex, brain nuclei, ganglion cells, and in many organs innervated by the ANS. For example, abnormal αSyn formed in the Auerbach/Meissner plexuses of the intestine would travel to the DMC along the vagal nerves, which show LBs/LNs and clear signs of atrophy in many cases of PD [10,134]. Brainstem nuclei connected to the DMC would subsequently be affected. This model is similar to the “body-first” model.

(c) Prefrontal–cortico-pontine PD, where the microvascular hypoperfusion is located to the prefrontal cortex, and pigmented neurons of the pons would be retrogradely degenerated as explained. Subsequently, misfolded αSyn would spread throughout the brain upward (substantia nigra, limbic system, neocortex) and downward (medulla oblongata, spinal cord, PNS). IRBD, cognitive impairment, and Lewy pathology in the periphery are frequently associated with this PD model.

(d) Amygdaline PD, where the amygdala is the site of microvascular flow disturbances. Abnormal αSyn formed in neurons of the amygdala would spread from this limbic structure throughout the brain, spinal cord and PNS.

## 6. Therapeutic Approaches

The proposed hypothesis may have implications for the identification of new therapeutic targets and early preventive strategies. Anti-inflammatory drugs, vascular stabilizing drugs, blockers of leukocyte infiltration into neural tissue, or MPO inhibitors should prevent PD or could be used in the early stages of the disease. In this regard, some preclinical and clinical studies have been conducted.

Non-steroidal anti-inflammatory drugs (NSAIDs) are currently being investigated in PD mainly as anti-inflammatory adjuncts rather than established disease-modifying therapies. Ongoing and completed trials registered on ClinicalTrials.gov have focused primarily on the selective COX-2 inhibitor celecoxib as an add-on to standard dopaminergic treatment [135,136]. A completed randomized controlled pilot study reported that six months of celecoxib added to levodopa/carbidopa improved motor scores and reduced circulating inflammatory markers and αSyn levels [137]. However, these findings derive from small, single-center studies and do not yet demonstrate long-term disease modification or preventive efficacy. Beyond interventional trials, epidemiological studies have explored whether NSAID exposure influences PD risk [138,139,140,141,142,143,144,145,146]. Among these, ibuprofen (but not aspirin or other NSAIDs) has shown the most consistent association with reduced Parkinson’s disease incidence. Meta-analyses and population-based studies, however, have yielded heterogeneous results, likely reflecting confounding factors such as indication, dosage, and duration of use. Overall, current clinical evidence supports further investigation of selective NSAIDs, particularly COX-2 inhibitors, as symptomatic or biomarker-modulating agents. At present, there is insufficient evidence to recommend NSAIDs for PD prevention. Given the well-known gastrointestinal, renal, and cardiovascular risks of NSAIDs, any preventive or disease-modifying use would require clear benefit demonstrated in adequately powered randomized trials.

Nilotinib, a drug that modulates systemic immune responses and inhibits leukocyte infiltration into affected tissue, improves motor behavior in PD models [147]. This drug has been tested in clinical trials with patients with advanced PD [148]. Nilotinib shows safety and good tolerability, as well as an improvement in some CSF biomarker levels (reduction in αSyn and increase in 3,4-dihydroxyphenylacetic acid), but it does not offer clinical advantages due to the lack of beneficial effects on motor deficits [148]. However, this drug has not been tested in patients with early-stage PD or as a preventive compound.

The administration of vascular stabilizing drugs together with leukocyte infiltration blockers may be an interesting approach. A recent experimental study using a combination of a blood vessel protector (angiopoietin-1) and an inflammatory cell infiltration blocker (C16) in a murine model of PD indicates that both drugs produce positive effects that attenuate the infiltration of immune cells and subsequent inflammation, reduce dopaminergic cell death, and improve functional disability [149]. Clinical studies should be conducted on the efficacy of this type of drug combination.

Regarding MPO, it has already been proposed that this enzyme be targeted for treatment of Parkinson’s disease [100,150,151]. In a trial with AZD3241, a selective MPO inhibitor, Jucaite and colleagues have provided evidence supporting the drug’s mechanisms of action in PD patients [151]. However, the authors administered the drug for 8 weeks and acknowledge that longer treatment is required to determine whether it has beneficial effects for therapy. MPO inhibitors have not been tested for the prevention of PD. Finally, the hypothesis may help identify vascular and/or immune biomarkers related to the altered vascular-immune-oxidative axis proposed here.

## 7. Conclusions

Early detection of microvascular flow alterations or lesions and αSyn deposits (e.g., using MRI or SPECT) at any of the “critical points” in the nervous system could be useful for the prevention of idiopathic Parkinson’s disease. The proposed hypothesis could have implications for the identification of new therapeutic targets, early prevention strategies, and the development of vascular and/or immune biomarkers. It is necessary to test the hypothesis that “microvascular flow disturbance can cause hypoperfusion and leukocyte infiltration at critical points in the nervous system characterized by the presence of pigmented cells.”

## Figures and Tables

**Table 1:** PD subtypes according to the site of microvascular flow disturbance.

PD Subtype	Original Microvascular Flow Disturbance
Olfactory epithelium/bulb PD	Olfactory epithelium
PNS/brain PD	Autonomic nervous system Enteric nervous system
Prefrontal–cortico-pontine PD	Prefrontal cortex
Amygdaline PD	Amygdala

Abbrev.: PD, Parkinson’s Disease; PNS, peripheral nervous system.

## Data Availability

The original contributions presented in this study are included in the article. Further inquiries can be directed to the corresponding author(s).

## Abbreviations

The following abbreviations are used in this manuscript:

ANS	Autonomic Nervous System
αSyn	alpha-synuclein
BBB	Blood–brain barrier
CFS	Cerebrospinal fluid
CNS	Central Nervous System
DMC	the dorsal motor nuclear complex of the glossopharyngeal and vagus nerves
ENS	Enteric Nervous System
IRBD	Isolated REM Sleep Behavior Disorder
LBs/LNs	Lewy bodies/Lewy neurites
LF	Lipofuscin
MPO	Myeloperoxidase
MRI	Magnetic Resonance Imaging
NM	Neuromelanin
PD	Parkinson’s disease
PNS	Peripheral Nervous System
SPECT	Single photon emission computed tomography
